# Otorhinolaryngological Management in Taiwanese Patients with Mucopolysaccharidoses

**DOI:** 10.7150/ijms.61827

**Published:** 2021-07-25

**Authors:** Chung-Lin Lee, Kuo-Sheng Lee, Chih-Kuang Chuang, Chin-Hui Su, Huei-Ching Chiu, Ru-Yi Tu, Yun-Ting Lo, Ya-Hui Chang, Hsiang-Yu Lin, Shuan-Pei Lin

**Affiliations:** 1 Department of Pediatrics, MacKay Memorial Hospital, Taipei, Taiwan; 2 Institute of Clinical Medicine, National Yang-Ming University, Taipei, Taiwan; 3 Institute of Clinical Medicine, National Yang-Ming Chiao Tung University, Taipei, Taiwan; 4 Department of Medicine, MacKay Medical College, New Taipei City, Taiwan; 5 MacKay Junior College of Medicine, Nursing and Management, Taipei, Taiwan; 6 Department of Rare Disease Center, MacKay Memorial Hospital, Taipei, Taiwan; 7 Department of Otorhinolaryngology and Head & Neck Surgery, MacKay Memorial Hospital, Taipei, Taiwan; 8 Division of Genetics and Metabolism, Department of Medical Research, MacKay Memorial Hospital, Taipei, Taiwan; 9 College of Medicine, Fu-Jen Catholic University, Taipei, Taiwan; 10 Department of Medical Research, China Medical University Hospital, China Medical University, Taichung, Taiwan; 11 Department of Infant and Child Care, National Taipei University of Nursing and Health Sciences, Taipei, Taiwan.

**Keywords:** Adenotonsillectomy, tympanostomy, mucopolysaccharidoses, obstructive sleep apnea syndrome, otorhinolaryngological, Taiwan

## Abstract

**Background:** Mucopolysaccharidoses (MPSs) are lysosomal storage disorders wherein glycosaminoglycans accumulate because the enzymes that degrade them are insufficient. The earliest symptoms, which are the main reasons for seeking consultation, are otorhinolaryngological and commonly occur in MPS I, II, IV, and VI. This retrospective study aimed to determine the occurrence of otorhinolaryngological manifestations in MPS patients in Taiwan and to analyze the prognosis of surgical intervention, including its effect on symptoms.

**Methods:** We reviewed 42 patients (30 males and 12 females), with a median age of 20.5 years, who had MPS (16.7% type I, 35.7% type II, 19.0% type IIIB, 21.4% type IVA, and 7.2% type VI). The following otorhinolaryngological manifestations were collected: annual number of upper respiratory tract infections (URTIs) and otitis media with effusion (OME) episodes, adenoid size, tonsillar size, and apnea-hypopnea index (AHI).

**Results:** Among 42 patients, we found recurrent otitis media in 42.9% of the patients, hearing loss in 83.3% (mixed: 52.4%, conductive: 21.4%, and sensorineural: 9.5%), frequent URTIs in 47.6%, and obstructive sleep apnea syndrome in 35.7%. Moreover, 76% of the patients underwent ear, nose, and throat (ENT) surgery, including adenoidectomy, tonsillectomy, tympanostomy with ventilation tube insertion, tracheotomy, and supraglottoplasty.

**Conclusions:** MPS patients had a high incidence of ENT problems. ENT surgery reduced the severity of hearing loss, degree of symptoms related to upper airway obstruction, and severity of respiratory tract and otological infections of patients with MPS.

## Introduction

Mucopolysaccharidoses (MPSs) are a group of lysosomal storage disorders, classified into seven types (I, II, III, IV, VI, VII, and IX) 1,2. Most of their inheritance patterns are autosomal recessive, except for MPS type II, which is X-linked recessive. MPSs are caused by the deficiency of the enzymes that break down glycosaminoglycans (GAGs). Owing to the accumulation of GAGs in lysosomes, dysfunction of cells, tissues, and organs occurs. This results in coarse facial features, hepatosplenomegaly, bone deformities with limitation of joint movement, variable intellectual disability, cardiac anomalies, and corneal clouding 3,4.

Otorhinolaryngological manifestations frequently occur in MPS I, II, IV, and VI and are often the earliest clinical manifestations of these diseases 5,6. The abnormal accumulation of GAGs in the middle ear mucosa, nasal mucosa, and Eustachian tubes can lead to potential of stiffness and obstruction of the Eustachian tube 6. MPS patients typically have otitis media with effusion (OME), which could cause conductive hearing loss 7,8. It is also believed that infiltration of GAGs into the cochlear nerve, afferent cochlear nerve, and stria vascularis in the cochlea can cause sensorineural hearing loss 5. However, most hearing loss patterns of MPS VI patients are conductive 9, suggesting that the Eustachian tubes are involved in the mechanism of conductive hearing loss. Other ear, nose, and throat (ENT) disorders such as persistent copious nasal discharge, chronic recurrent rhinitis 10, and adenotonsillar hypertrophy often occur in MPS patients as well 7.

Upper airway complications and obstructive sleep apnea (OSA) can be caused by tonsilloadenoid hypertrophy, short and stiff neck, macroglossia and stiffness of the oropharynx, temporomandibular joint stiffness, nasal dysmorphism, flaccid and redundant supra-arytenoid soft tissue, and tracheomalacia 9-12. Even though obstructed airways can be improved by conservative treatment such as positive airway pressure devices, there may still be a need for early adenotonsillectomy and even tracheostomy 7,13. This retrospective study aimed to determine the occurrence of ENT manifestations in MPS patients in Taiwan and to analyze the prognosis of surgical intervention, including the effect of surgeries on symptoms.

## Materials and Methods

### Ethical compliance

This study was approved by the Ethics Committee of MacKay Memorial Hospital in Taipei, Taiwan.

### Study population

We reviewed the data of 42 MPS patients at the Department of Pediatrics and Otorhinolaryngology, MacKay Memorial Hospital, Taipei between January 2010 and December 2020. The data were categorized as MPS I (7 patients, 16.7%), MPS II (15 patients, 35.7%), MPS IIIB (8 patients, 19.0%), MPS IVA (9 patients, 21.4%), and MPS VI (3 patients, 7.2%). There were 30 males and 12 females with a median age of 20.5 years (range: 5-40). All patients consulted an otorhinolaryngologist regarding the need for surgery at a median age of 5.5 years. All patients, except those with MPS III, were receiving enzyme replacement therapy (ERT) and were alive at the time of study.

### Patient evaluations

Besides polysomnography (PSG), cooperative patients underwent flexible laryngobronchoscopy, sinoscopy, otoscopy, tympanograms, and audiometry. We evaluated them for the following: (1) annual number of upper respiratory tract infections (URTIs) and OME episodes; (2) degree of adenoid size (based on flexible nasopharyngoscopic examination, Grade 1: none of the adjacent structures such as the vomer, soft palate, and torus tubaris are in contact with the adenoid tissue; Grade 2: adenoid tissue is in contact with the torus tubaris; Grade 3: adenoid tissue is in contact with the torus tubaris and vomer; Grade 4: adenoid tissue is in contact with the torus tubaris, vomer, and soft palate at rest) 14; (3) degree of tonsillar size (Grade 0: absence of tonsillar tissue; Grade 1: within the pillars; Grade 2: extended to the pillars; Grade 3: extended past the pillars; Grade 4: extended to the midline) 15; and (4) the apnea-hypopnea index (AHI, number of obstructive apnea and hypopnea events per hour of sleep) to identify obstructive sleep apnea syndrome (OSAS) if AHI > 5 in adults and > 1 in children.

We also used the infection score system to evaluate the severity of respiratory tract and otological infections 16. This includes an evaluation of the type of infection, systemic symptoms, daily activity, therapy, hospitalization, and resolution time (Table 1). Total scores of ≤5, 6-11, and 12 indicate mild, moderate, and severe respiratory tract and/or otological infection, respectively. SPSS version 25.0 (SPSS, Inc., Chicago, IL) was used to perform the statistical analysis. Statistical significance was set at *p* < 0.05. The study protocol was approved by the Ethics Committee of MacKay Memorial Hospital, and written informed consent was provided by parents of patients under 18 years of age and from the patients themselves if they were over 18 years old.

## Results

Of the 42 MPS patients, 32 (76.2%) underwent surgery (Table 2), including adenotonsillectomy (20 patients, 47.6%), adenoidectomy only (0 patients, 0.0%), tonsillectomy only (2 patient, 4.8%), insertion of middle ear ventilation tubes (24 patients, 57.1%), tracheotomy (2 patients, 4.8%), and CO_2_ laser supraglottoplasty (1 patient, total two times, 2.4%). All patients had at least one ENT symptom (Table 3). We also found that 15 patients (35.7%) had a history of chronic and recurrent OME (≥5 episodes) in a year.

Hearing loss, seen on pure tone audiometry, was noted in 35 patients (83.3%; 5 in MPS I, 15 in MPS II, 4 in MPS IIIB, 8 in MPS IVA, and 3 in MPS VI). Specifically, 22 patients (52.4%) had mixed-type hearing loss, 9 (21.4%) had conductive hearing loss, and 4 (9.5%) had sensorineural hearing loss. Among 20 patients analyzed using PSG, 16 (80.0%) were diagnosed with OSA, and 15 (75.0%) underwent tonsillectomy or adenotonsillectomy. AHI ranged from 0 to 83.1.

**Figure 1:**
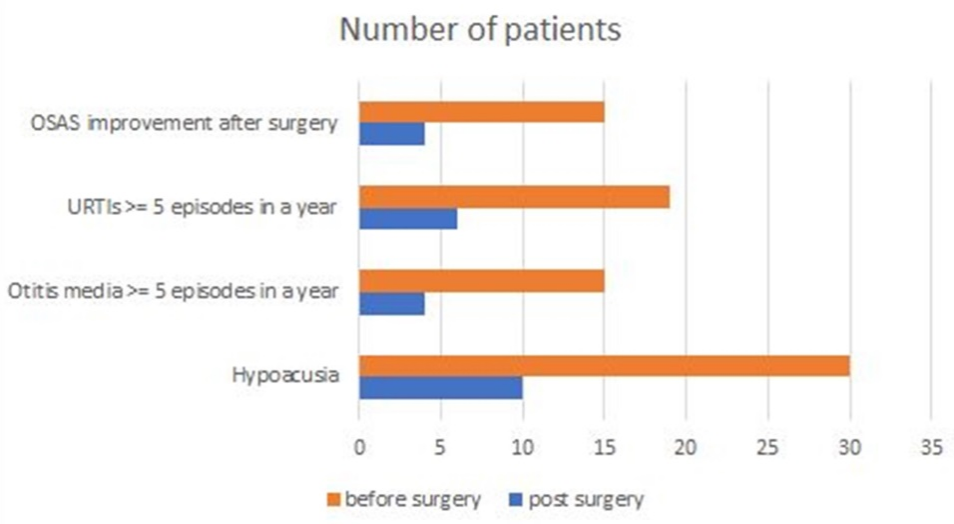
Otorhinolaryngological manifestations of patients with mucopolysaccharidoses before and after surgery. OSAS: obstructive sleep apnea syndrome; OSAS improvement after surgery: patients with a decrease in apnea-hypopnea index (AHI) by more than 50% after surgery; URTIs: upper respiratory tract infections

Figure 1 presents the comparison between ENT manifestations before and after surgery. According to the infection score system, improvements in the severity of respiratory symptoms and ENT infections were seen after surgery (mean infection score before vs. that after surgery: 5.8 ± 1.6 *vs*. 3.8 ± 0.9, *p* < 0.05). Out of 15 OSAS patients, 4 (26.7%) demonstrated a decrease in AHI by more than 50% after surgery. All patients were alive at the time of writing.

## Discussion

To the best of our knowledge, this is the first report to describe the otorhinolaryngological management of Taiwanese patients with MPS. Our results emphasize that otorhinolaryngological management is important for patients with MPS because they have problems in language development and poor quality of life due to the high frequency of ear disorders 17. Otorhinolaryngologists and audiologists play important roles in the follow-up and treatment of MPS patients 8. Patients need better and long-term follow-up because of the high incidence of recurrent serous otitis media with conductive hearing loss and progress to sensorineural hearing loss.

In previous studies, an average of 75% of MPS cases (range: 59.7%-89%) have hearing loss 18. Various types and degrees of hearing loss can be seen in MPS patients 7,8,19-21. Conductive hearing losses are the most common in MPS patients because of frequent chronic middle ear effusion and Eustachian tube dysfunction. However, the incidence and etiology of sensorineural hearing losses are unknown 7. Similarly, our study found that conductive hearing losses were more common than sensorineural hearing losses (21.4% vs. 9.5%). Although conductive hearing loss can be improved by adenoidectomy and tympanostomy with ventilation tube insertion 21, sensorineural hearing loss remains a problem to be overcome. In our study, 34.3% of patients had improved hearing after surgery. Furthermore, according to the infection score system, we noticed a decreased severity of respiratory tract and otological infections after ENT surgery. This may be due to the improvements in adenoid size, tonsillar size, and OME with decreased infection risk. Our local guidelines recommend audiometry assessment for all MPS cases on an annual basis. Conductive hearing loss is common in MPS patients in the early stage. Patients would develop either sensorineural or mixed-type hearing loss afterward. Thus, the early diagnosis of conductive hearing impairment and its treatment is likely to improve quality of life before progression to sensorineural or mixed-type hearing loss.

Ventilation tubes are advised for MPS patients with recurrent or persistent OME and hearing loss. However, some families refused the insertion of ventilation tubes because they were afraid of the risk of general anesthesia during operation. In some cases, the operation was very difficult or impossible because severe deformity of the external ear canal made the ear drum difficult to approach. In some cases, we advised patients to use hearing aids, but financial difficulties made it difficult for them to come to follow-ups and obtain these aids. This is the reason why some patients still have conductive hearing loss after ERT. Besides that, ERT could not improve sensorineural hearing loss 22.

Upper airway obstruction can cause serious morbidity and mortality. Most respiratory problems are caused by soft tissue changes of the tonsils, adenoids, tongue, and lingual tonsils and by the stiffness of the oropharynx and temporomandibular joint. Oropharyngeal stiffness and collapse become severe when the disease deteriorates; this can cause significant airway obstruction 23. The degree of upper airway obstruction may range from OSA to life-threatening airway emergencies, and airway evaluation is thus necessary but challenging. The results of airway examinations vary between patients 24. In our study, the rate of upper airway obstruction (patients who had stridor, suprasternal retractions, and change of voice) was 76.2%, compared to 38% 20, 48% 18, and 92% 25 in other studies. All types of MPS patients had similar symptoms such as stridor, suprasternal retractions, and voice change. Consequently, it is necessary to perform adenoidectomy in MPS patients with purulent, recurrent, and chronic symptoms such as OME, snoring, and sleep apnea 14. Though tonsillectomy and adenoidectomy can help those with OSA at first, they may need nocturnal oxygen treatment and even tracheostomy in advanced cases 26. Moreover, patients with MPS have greater anesthetic risks because they have macroglossia, temporomandibular joint stiffness, difficult or failed intubations, abnormal laryngeal anatomy, trachea deformity, and subglottic narrowing 27,28. Before surgery, these patients need examination using a flexible bronchoscope to survey the exact extent and severity of airway obstruction 7,9.

Patient history and physical examination are necessary for the initial evaluation of OSA, but the degree of obstruction before and after surgery should be studied using PSG and laryngobronchoscopy 25,29. In our study, 11 out of 15 OSA patients (73.3%) had no apparent improvement (i.e., AHI did not decrease by >50% after surgery) after adenotonsillectomy due to macroglossia and oropharyngeal stiffness. This condition was also found in other OSA patients without MPS. This may be because even though the structure and tension of the upper airway improved after surgery, persistent stiffness of the oropharynx and macroglossia could deteriorate after years. Thus, ENT care and entire airway fiberendoscopy evaluations are important before operating on such patients; these could lead to safer intubation and extubation. Choosing a suitable size of endotracheal tube and the method of intubation can potentially decrease the risk of complications related to intubation during general anesthesia and surgery 30.

According to Stepien et al. 31, because the standard tools and assessments used by anesthesiologists may not be adequate for the assessment of MPS patients with complex airways, a more thorough assessment involving an ENT consultant should also be carried out preoperatively. In our center, before giving general anesthesia to MPS patients, entire airway evaluation with flexible fiberbronchoscopy is always done beforehand. We then decide and discuss with an anesthesiologist regarding the type of anesthesia and size of the endotracheal tube. In our clinical practice, the tracheal tube is changed monthly by ENT doctors either in a sitting position (in the ENT outpatient department) or in the supine position (in the ward). In a sitting position, the airway is more patent, making it more convenient to change the tracheal tube than in the supine position. Nevertheless, 4% lidocaine may still be sprayed into the tracheal tube before changing the tracheal tube. Flexible bronchoscopy is then performed after changing the tracheal tube to check the position of the top of the tracheal tube. There were no airway emergencies in our previous procedures.

Tracheostomy in MPS patient typically needs an adjustable tracheal tube to stent the entire tracheal length. We use the Bivona® adjustable tracheal tube (Smith Medical, https://www.smiths-medical.com/area-of-care/homecare-for-clinical/bivona-tracheostomy) to stent the entire tracheal length for the tracheostomy tube. MPS patients typically have deformed and narrow tracheal lumens; thus, smaller, adjustable tracheal tubes are needed. However, these tubes are not long enough to stent the entire tracheal length. Because of this, we need a longer, custom-made tracheal tube to stent the entire trachea.

This study has several limitations. We had only 42 patients in this study because they were the only ones with complete medical histories of otorhinolaryngological treatments. Not all patients had PSG results in our study. All MPS patients should have an overnight study performed at least once in childhood and repeated every 2-3 years depending on the initial results and their symptoms. The quality of life of patients should have also been formally evaluated using the visual analog scale as done in a previous study 16.

## Conclusion

The high incidence of ENT problems in MPS patients reinforces that ENT surgery remains a fundamental treatment modality for resolving OME, improving hearing acuity, and relieving upper airway obstruction. ENT surgery can decrease the severity of the respiratory tract and otological infections of patients with MPS.

## Figures and Tables

**Table 1:** Severity of respiratory tract and otological infections of patients with mucopolysaccharidoses evaluated using the infection score system

Score		
**Type of infection**	Rhinitis	0
	Rhinitis + otitis and/or tonsillitis	1
	Pneumonia	2
**Systemic symptoms**	Absent	0
	Slight fever and/or some aches	1
	Definite elevation of temperature	2
**Daily activity**	Not limited	0
	Some limitation	1
	Severely incapacitated	2
**Therapy**	Local	0
	Systemic (oral administration)	1
	Systemic (intravenous administration)	2
**Hospitalization**	No	0
	Single entry followed by home therapy	1
	Admission	2
**Resolution**	<7 days	0
	7-10 days	1
	>10 days	2

Total scores of ≤5, 6-11, and 12 indicate mild, moderate, and severe respiratory tract and/or otological infection, respectively.

**Table 2:** Surgical procedures performed

Patients	Adenoidectomy	Tonsillectomy		Adenotonsillectomy		Insertion of middle ear ventilation tubes		Tracheotomy	Laser supraglottoplasty	
2										
4					x		x	x		
5					x		x			
6					x					
7							x			
8			x				x			
9					x		x			
10							x			
12							x			
14					x		x			
15					x					
16					x		x			
17					x		x			
18					x		x			
19					x		x			
20							x			
21					x		x			
22					x		x			
23					x					
25							x			
26					x		x			
30					x		x			
31					x					
33					x		x			
35										x
36							x			
37			x				x			
38					x					
39								x		
40					x		x			
41					x		x			
42							x			
										

**Table 3:** Otorhinolaryngological manifestations of patients with mucopolysaccharidoses before and after ENT surgery.

Patient	MPS	Age	Gender	Numbers of otitis mediaepisodesper year	Hypoacusia	Degree of adenoidsize before surgery	Degree of adenoid size after surgery	Degree of tonsillarsize before surgery	Degree of tonsillar size after surgery	Numbers of URTIepisodesper year	OSASAHI before surgery	OSASAHI after surgery	AHI improve > 50% after surgery
1	I	39	male	3	sensorineural	1	No surgery	0	No surgery	2	46.2	29.1	No
2	I	28	female	5	normal	2	1	0	0	5	None	None	None
3	I	40	male	2	sensorineural	1	No surgery	0	No surgery	4	10.1	9.5	No
4	I	7	female	7	mixed	4	1	2	0	6	None	None	None
5	I	7	female	6	mixed	4	1	2	0	5	None	None	None
6	I	19	male	2	normal	2	0	2	0	1	49.8	32.8	No
7	I	21	male	3	conductive	4	1	3	0	3	15.0	14.2	No
8	II	26	male	1	mixed	2	0	2	0	3	None	None	None
9	II	23	male	5	mixed	3	0	2	0	7	None	None	None
10	II	29	male	3	mixed	2	0	1	0	1	None	None	None
11	II	27	male	1	mixed	1	No surgery	1	No surgery	2	None	None	None
12	II	31	male	3	mixed	2	0	2	0	3	56.1	37.3	No
13	II	26	male	4	mixed	1	No surgery	1	No surgery	3	None	None	None
14	II	16	male	6	mixed	3	1	3	0	6	None	None	None
15	II	26	male	8	mixed	3	1	3	0	8	43.1	11.1	Yes
16	II	13	male	4	mixed	4	1	3	0	7	None	None	None
17	II	8	male	6	conductive	4	1	3	0	6	None	None	None
18	II	8	female	2	mixed	3	0	3	0	1	None	None	None
19	II	6	male	4	conductive	3	0	3	0	3	2	1.4	No
20	II	17	male	7	conductive	3	1	2	0	9	None	None	None
21	II	7	male	1	mixed	2	0	2	0	1	None	None	None
22	II	5	male	6	mixed	4	1	3	0	8	39	13	Yes
23	IIIB	8	male	1	mixed	4	0	3	0	2	None	None	None
24	IIIB	7	female	4	normal	1	No surgery	0	No surgery	2	None	None	None
25	IIIB	11	male	8	mixed	2	0	1	0	1	None	None	None
26	IIIB	6	female	6	mixed	4	1	3	0	8	None	None	None
27	IIIB	24	female	2	normal	1	No surgery	1	No surgery	3	None	None	None
28	IIIB	24	female	1	normal	1	No surgery	1	No surgery	2	None	None	None
29	IIIB	6	male	3	normal	4	No surgery	2	No surgery	3	None	None	None
30	IIIB	7	female	1	mixed	4	1	3	0	4	5.3	4.9	No
31	IVA	34	male	3	sensorineural	3	0	2	0	6	26.2	24.9	No
32	IVA	10	male	2	conductive	4	No surgery	2	No surgery	5	3.1	None	None
33	IVA	25	female	7	mixed	3	0	2	0	8	None	None	None
34	IVA	9	male	0	sensorineural	3	No surgery	2	No surgery	9	None	None	None
35	IVA	32	male	1	conductive	1	0	0	0	5	None	None	None
36	IVA	22	male	0	mixed	2	0	2	0	6	None	None	None
37	IVA	15	male	2	mixed	2	1	3	0	7	8.5	4.2	Yes
38	IVA	20	female	1	normal	3	1	4	0	1	None	None	None
39	IVA	30	male	1	conductive	3	1	2	0	8	45.9	10.8	Yes
40	VI	21	male	5	conductive	4	1	4	0	7	55.8	51.8	No
41	VI	22	male	7	mixed	3	0	3	0	6	83.1	66.6	No
42	VI	27	female	6	conductive	2	0	1	0	1	19.7	17.6	No

MPS: mucopolysaccharidoses, URTI: upper respiratory tract infection; OSAS: obstructive sleep apnea syndrome; AHI: apnea-hypopnea index.
